# 557. Cost-effectiveness of Introducing Metagenomic Next-generation Sequencing (mNGS) for the Diagnosis of Suspected Neurological Infections Patients in China

**DOI:** 10.1093/ofid/ofad500.626

**Published:** 2023-11-27

**Authors:** Siyang Peng, Rui Liang, Peng Liu, Long Hu, Yaping Ai, Junfeng Wang, Guanqi Hong, Shuli Qu

**Affiliations:** Illumina, San Diego, California; Illumina, San Diego, California; Illumina, San Diego, California; Hugo Biotech, Beijing, Beijing, China; IQVIA, Shanghai, Shanghai, China; IQVIA, Shanghai, Shanghai, China; IQVIA, Shanghai, Shanghai, China; IQVIA, Shanghai, Shanghai, China

## Abstract

**Background:**

Early identification of neurological pathogens is crucial for favorable clinical outcomes. Metagenomic next-generation sequencing (mNGS) offers an alternative approach to identify pathogens in complex clinical samples. This study aimed to assess the cost-effectiveness of using mNGS in combination with traditional methods in diagnosing suspected neurological infections patients.

**Methods:**

A decision tree model was developed to illustrate the clinical pathway for patients with suspected neurological infections in China. Two strategies were compared, one utilizing mNGS combined with traditional pathogen detection methods, and the other relying solely on traditional methods including cerebrospinal fluid microbial culture, smear microscopy, and serological tests. The study was conducted from the Chinese healthcare system perspective. Survival outcome, life years outcome and cost outcomes were captured over a lifetime horizon. Model inputs, including cost (specimen collection cost, diagnosis cost, imaging examination cost, drug cost and inpatient bed cost), treatment efficacy, mortality, sensitivity and specificity of different testing strategies, were derived from published literature and experts’ interviews. Uncertainty was assessed by sensitivity analyses.

**Results:**

The base case analysis suggested that, compared with traditional pathogen detection methods, mNGS combined with traditional methods resulted in longer life-year (average 0.04 life-year gain) and lower cost (average saving of 6,419 CNY (947 USD)), among all patients tested. The life-years gain was primarily driven by the more accurate diagnosis and the consequent administration of more effective etiological treatment. The cost saving was largely driven by the reduction of drug costs which resulted from precision medicine-based treatment, instead of empirical treatment. The probabilistic sensitivity analysis confirmed the robustness of the model.

**Conclusion:**

Compared to traditional methods only, mNGS combined with traditional methods is a cost-effectiveness method for improving patient clinical outcomes while reducing healthcare costs.

**Disclosures:**

**All Authors**: No reported disclosures

